# Left atrial longitudinal strain by speckle tracking as independent predictor of recurrence after electrical cardioversion in persistent and long standing persistent non-valvular atrial fibrillation

**DOI:** 10.1007/s10554-019-01597-7

**Published:** 2019-04-16

**Authors:** Luis Antonio Moreno-Ruiz, Alejandra Madrid-Miller, Jerónimo Enrique Martínez-Flores, Jesús Antonio González-Hermosillo, Jorge Arenas-Fonseca, Noé Zamorano-Velázquez, Beatriz Mendoza-Pérez

**Affiliations:** 1 Division of Cardiology, UMAE Hospital de Cardiología, Centro Médico Nacional Siglo XXI, IMSS, 330 Cuauhtémoc Av, Doctores, Cuauhtémoc, 06720 Mexico City, Mexico; 2 Direction of Education and Research, UMAE Hospital de Cardiología, Centro Médico Nacional Siglo XXI, IMSS, 330 Cuauhtémoc Av, Doctores, Cuauhtémoc, 06720 Mexico City, Mexico; 3 Department of Electrophysiology, UMAE Hospital de Cardiología, Centro Médico Nacional Siglo XXI, IMSS, 330 Cuauhtémoc Av, Doctores, Cuauhtémoc, 06720 Mexico City, Mexico; 4 Medical Subadrees of Innovation and Development Project, Instituto Nacional de Cardiología “Ignacio Chávez”, 1 Juan Badiano, Sección XVI, Tlalpan, 14080 Mexico City, Mexico; 5 Department of Echocardiography, UMAE Hospital de Cardiología, Centro Médico Nacional Siglo XXI, IMSS, 330 Cuauhtémoc Av, Doctores, Cuauhtémoc, 06720 Mexico City, Mexico

**Keywords:** Atrial fibrillation, Longitudinal atrial strain, Recurrence

## Abstract

Atrial fibrillation (AF) is the most common arrhythmia in humans. After successful cardioversion, there is a recurrence of 60% due to atrial remodeling, and it has been shown that the global peak atrial longitudinal strain (GPALS) is decreased in these subjects. The aim of this study was to evaluate the predictive value of GPALS for AF recurrence. A prospective cohort of patients with persistent (PnVAF) and long standing persistent non-valvular AF (LSPnVAF) which underwent electrical cardioversion was evaluated with standard echocardiographic variables and GPALS quantification. The primary endpoint was AF recurrence at 6 months. We included PnVAF (n = 50, aged 68.4 ± 10.2 years, female 46%, lasted AF 6 months) and LSPnVAF (n = 81, aged 66.5 ± 13.1 years, female 36%, lasted AF 18 months). At 6 months there were a 68% of recurrence of AF in PnVAF and 53% in LSPnVAF group. GPALS was lower in recurrence 7.8 ± 2.0% versus 21.2 ± 8.9% (p < 0.001) for PnVAF and 7.3 ± 2.7% versus 20.7 ± 7.6% (p < 0.001) in LSPnVAF. GPALS ≤ 10.75% discriminates recurrence at 6 months with a sensitivity of 85%, specificity 99%, PPV 85%, NPV 90%, LR + 8.5 and LR- 0.17. The independent predictors of recurrence in PnVAF were GPALS ≤ 10.75% HR 8.89 [(2.2–35.7), p < 0.01] meanwhile in LSPnVAF were age HR 1.039 [(1.007-1.071), p = 0.01], and GPALS ≤ 10.75% HR 28.1 [(7.2–109.1), p < 0.001]. In subjects with PnVAF and LSPnVAF with successful electrical cardioversion, GPALS ≤ 10.75% predicts arrhythmia recurrence at 6-month follow-up.

## Introduction

Atrial fibrillation (AF) is the most frequent sustained arrhythmia in humans, and it is highly relevant due to the impact of the complications it produces. It can be treated by controlling the rhythm or the frequency [1–10]. Cardioversion is widely recommended for symptomatic subjects, even when receiving optimal treatment, and restores the physiological conditions of the atrium [8–14]. Recurrence after cardioversion reaches up to 60% in the first 12 months, a fact that has been associated to atrial remodeling [15–22]. Several authors consider the following as predictors of recurrence: age, use of angiotensin-converting enzyme inhibitors (ACE inhibitors), angiotensin II receptor blockers (ARBs), spironolactone, comorbidities, time of evolution of AF, increased diameter and atrial volumes, and low peak velocity of the left atrial appendage, among others [23–33]. Echocardiographic techniques as speckle tracking permit evaluate the structure and the function at a finer level thanks to the measuring of myocardial fiber deformation [34–40]; this technique allows measuring the global peak atrial longitudinal strain (GPALS), which decreases in subjects with AF and holds an inverse correlation with the degree of fibrosis measured by magnetic resonance and histology [41–44]. The aim of this study was to evaluate the predictive value of the GPALS in the discrimination of post-cardioversion arrhythmia recurrence in subjects with persistent non-valvular atrial fibrillation (PnVAF) and long standing persistent non-valvular AF (LSPnVAF).

## Materials and methods

From June 2015 to January 2018, we recruited subjects with PnVAF (AF that lasts longer than 7 days, including episodes that are terminated by cardioversion, either with drugs or by direct current cardioversion, after 7 days or more) and LSPnVAF (continuous AF lasting for ≥ 1 year when it is decided to adopt a rhythm control strategy) eligible for elective electrical cardioversion, at the Hospital de Cardiología, CMN Siglo XXI. The non-inclusion criteria were: pregnancy, ischemic disease, cardiomyopathy, congenital heart diseases, pulmonary artery systolic pressure (PASP) > 60 mmHg or left ventricular ejection fraction (LVEF) < 50%, need for emergency electrical cardioversion, previous cardioversion performed less than 6 months before, presence of single or dual-chamber pacemaker and average ventricular response > 110 beats per minute (bpm) or < 40 bpm. Four subjects were excluded due to poor echocardiography window and the inability to quantify the GPALS using QLAB software (Philips). The two-dimensional echocardiograms in DICOM format were analyzed to quantify the GPALS in 131 subjects. We also analyzed the demographic and echocardiographic conventional data, both transthoracic and transesophageal. All the subjects signed written informed consents to use the documentary studies and for clinical and phone follow-up. The study protocol was authorized for its implementation by the Local Research and Health Research Ethics Committee.

During the visit previous to cardioversion, demographic, clinical and anthropometric data were collected. The classification of type of AF was conducted by a trained electrophysiologist according to the interview and electrocardiographic evidence. The electrical cardioversion was carried out by the treating cardiologist, having ensured three previous weeks of optimal anticoagulation and no evidence of thrombus by transesophageal echocardiogram on the same day the procedure took place. We considered the cardioversion successful after the termination of AF was confirmed by the presence of organized atrial activity and criteria of normal sinus rhythm. All the subjects received 150 mg propafenone TID and metoprolol BID at titrated dose as maintenance treatment. The subjects were given clinical follow-up during the first four weeks; afterwards, the follow-up took place once a month including clinical and electrocardiographic assessment for 6 months. A Holter monitoring test was performed after one and 6 months or before in case the subject showed palpitations, collapse, dyspnea or thoracic pain in order to find AF recurrence. The presence of two or more episodes of irregular atrial activity lasting at least 10 min documented by Holter monitoring or ECG was considered recurrence after a successful cardioversion. Two researchers, a cardiologist and an electrophysiologist, with independent blinded quality, verified these episodes.

**Fig. 1:**
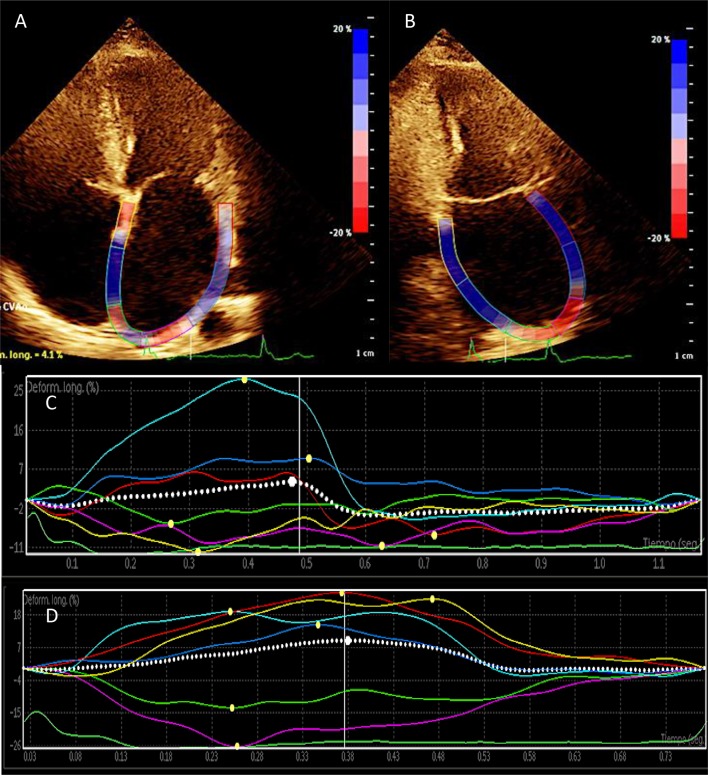
Representative global peak atrial longitudinal strain (GPALS) images. Apical long axes of four (**a**) and two chambers (**b**) with tracing the atrial endocardial border. Automated GPALS quantification with two-dimensional speckle tracking in apical views of four (**c**) and two chambers (**d**). We defined GPALS as the average of three beats each, in this case GPALS was 6.8% in a subject with AF recurrence. *GPALS* global peak atrial longitudinal strain, *AF* atrial fibrillation

All the transthoracic echocardiography studies were conducted placing the subject lying down on the back or left side, using a Philips iE33 commercial ultrasound system with S5-1 multi frequency transducer, following the recommendations provided by the American Society of Echocardiography. We obtained standard two-dimensional images of the cardiac chambers, valves and great vessels from the parasternal and apical long axes (4, 2 and 3 chambers) with a speed of 80–100 frames per second, including three beats per registry and simultaneous electrocardiography monitoring. We measured the length and volume of both atria and aortic annulus after systole, while the aortic root and dimensions and parietal width of both ventricles were measured at the end of ventricular diastole. We calculated the ejection fraction by modified Simpson’s method, shortening fraction and final systolic and diastolic ventricular volumes. From the apical four-chamber view, we obtained the spectral registry of the mitral inflow pattern using pulsed Doppler. We measured the early diastolic filling velocity (E-wave), its deceleration (DT), and the isovolumic relaxation time (IVRT). In addition, we obtained the velocity of e’ and S using pulsed Doppler to calculate the E/e’ ratio, after calculating the average of the values from the mitral septal-lateral annulus. We determined the area of the atria in systole from the four-chamber view. In order to determine the atrial volumes, we applied the area-length method in views of four and two chambers in systole. Once they were averaged, they were indexed to the body surface (Mosteller formula). We registered apical views of four and two chambers in sequences of three beats each for outboard analysis. We took the velocity of the left appendage with pulsed Doppler from the transesophageal echocardiogram performed during the usual assessment protocol using an S7-2 omni probe (Philips). The GPALS outboard analysis was done using QLAB Advanced Quantification Software version 8.1 (Philips) with two-dimensional speckle tracking by two independent and blinded echocardiographers. The GPALS calculation was done tracing the atrial endocardial border while its automated determination was completed using QLAB (Philips) in the apical views of four and two chambers (Fig. 1), with an average of three beats each; we calculated the average of the values to obtain the value of the GPALS.

### Statistical analysis

We performed an exploratory analysis including the Kolmogorov–Smirnov test to determine the type of data distribution. The continuous variables were described according to their distribution (average ± standard deviation, mean and range) while the qualitative ones were expressed as frequencies and percentages. We used Student’s t test or the *U* Mann–Whitney test according to the distribution for differences between groups of quantitative variables and Chi squared distribution for groups of qualitative variables. We assessed reproducibility by selecting ten random echocardiographic studies that were recorded as raw data for intra and interobserver variability. Observers were blinded; for intraobserver variability, the same observer evaluated raw echocardiographic data 1 month after the first evaluation. We performed a receiver-operating characteristic (ROC) curve to determine the cut-point of GPALS associated to recurrence. We calculated sensitivity, specificity, predictive values and the likelihood ration for the calculated cut-point. A multivariate Cox proportional hazards regression analysis was conducted to evaluate the effect of the potentially confounding variables, event-free (recurrence) survival curve and calculation of adjusted HR. The variables associated to recurrence p < 0.20 were introduced to the model; a value p ≤ 0.05 was considered significant. The statistical analysis was carried out using SPSS Statistics V21.0 (IBM Corporation).

## Results

The study sample included 50 subjects with PnVAF (aged 68.4 ± 10.2 years, female 46%, lasted AF 6 months) and 81 with LSPnVAF (aged 66.5 ± 13.1 years, female 36%, lasted AF 18 months). Baseline and demographic characteristics are summarized in Table 1.

**Table 1:** Demographic characteristics of the subjects

	PnVAF (n = 50)				LSPnVAF (n = 81)			
	Total (n = 50)	Sinus rhythm (n = 16)	AF recurrence (n = 34)	p value*	Total (n = 81)	Sinus rhythm (n = 38)	AF recurrence (n = 43)	p value*
Female, n (%)	18 (36)	6 (38)	12 (35)	0.88	37 (46)	20 (52)	17 (39)	0.23
Age (years)	68.4 ± 10.2	69.6 ± 8.9	67.9 ± 10.9	0.57	66.5 ± 13.1	63.7 ± 14.9	69.0 ± 10.9	0.12
Weight (kg)	73.5 ± 10.2	69.4 ± 8.3	75.5 ± 10.5	0.04	76.0 ± 11.5	75.0 ± 10.6	76.8 ± 12.3	0.47
Height (m)	1.60 ± 0.09	1.59 ± 0.09	1.61 ± 0.08	0.55	1.62 ± 0.09	1.62 ± 0.08	1.62 ± 0.10	0.93
BSA (m^2^)	1.79 ± 0.15	1.74 ± 0.13	1.82 ± 0.16	0.09	1.83 ± 0.18	1.81 ± 0.16	1.84 ± 0.20	0.49
BMI (kg/m^2^)	28.5 ± 3.9	27.4 ± 4.3	29 ± 3.6	0.16	28.6 ± 3.2	28.4 ± 3.5	28.8 ± 2.9	0.63
AF lasted (months)	6 (3–11)	5 (3–11)	6 (3–11)	0.20	18 (13–120)	18 (13–120)	18 (13–120)	0.58
Diabetes, n (%)	14 (28)	4 (25)	10 (29)	0.74	20 (25)	5 (13)	15 (34)	0.02
Hypertension, n (%)	47 (94)	14 (87)	33 (97)	0.23	68 (84)	30 (79)	38 (89)	0.24
Smokers, n (%)	17 (34)	5 (31)	12 (35)	0.77	29 (36)	16 (42)	13 (30)	0.26
Dyslipidemia, n (%)	9 (18)	3 (19)	6 (18)	0.92	18 (22)	12 (32)	6 (14)	0.06
ACEI, n (%)	17 (34)	6 (38)	11 (32)	0.72	33 (41)	15 (39)	18 (42)	0.82
ARBs, n (%)	18 (36)	4 (25)	14 (41)	0.26	30 (37)	15 (39)	15 (35)	0.66
Spironolactone, n (%)	5 (10)	1 (6)	4 (12)	0.55	10 (12)	5 (13)	5 (12)	0.83
Statins, n (%)	10 (20)	3 (19)	7 (21)	0.88	19 (23)	11 (29)	8 (19)	0.27

The continuous variables were described according to their distribution (mean ± standard deviation, mean and range) while the qualitative ones were expressed as frequencies and percentages

*PnVAF* persistent non-valvular atrial fibrillation, *LSPnVAF* long standing persistent non-valvular atrial fibrillation, *AF* atrial fibrillation, *BSA* body surface area, *BMI* body mass index, *ACEI* angiotensin-converting enzyme inhibitors, *ARBs* angiotensin II receptor blockers

*Chi squared, Fisher’s exact test, Student’s *t* test or U Mann–Whitney according to their distribution

At 6 months, the subjects with PnVAF showed 68% (n = 34) of recurrence after cardioversion versus 53% (n = 43) among the subjects with LSPnVAF (p = ns). The latter group showed earlier recurrence (14 vs. 30 days; p = 0.04). The analysis in the PnVAF group showed higher body weight among subjects with AF recurrence than those in sinus rhythm (75.5 ± 10.5 kg vs. 69.4 ± 8.3 kg; p = 0.04), there were no differences in terms of other demographic variables. In the LSPnVAF subjects there was a greater frequency of diabetes mellitus in the recurrence group (34 vs. 13%; p = 0.02) and also there were no differences in terms of other variables as shown in Table 1.

The subjects of PnVAF with AF recurrence showed lower velocity of left atrial appendage (18.3 ± 4.3 vs. 22.1 ± 5.5 cm/s), but there were no differences in left atrial diameters, volumes and area. In the LSPnVAF group the subjects with AF recurrence showed greater antero-posterior (45.9 ± 5.6 vs. 42.5 ± 5.8 mm; p < 0.01), and cephalocaudal diameter (62.7 ± 7.1 vs. 50.9 ± 8.3 mm; p < 0.001), left atrial volume (73.1 ± 20.6 vs. 57.2 ± 21.2 ml; p = 0.001), indexed volume (41.1 ± 14.2 vs. 32.4 ± 12.1 ml/m^2^; p < 0.01) and area (25.7 ± 6.6 vs. 21.6 ± 5.9 cm^2^; p < 0.01), but lower velocity of the left atrial appendage (18.3 ± 3.9 vs. 21.8 ± 5.5 cm/s; p < 0.01) when compared to the sinus rhythm subjects as shown in Table 2.

**Table 2:** Echocardiographic characteristics of the left atrium

	PnVAF (n = 50)				LSPnVAF (n = 81)			
	Total (n = 50)	Sinus rhythm (n = 16)	AF recurrence (n = 34)	p value *	Total (n = 81)	Sinus rhythm (n = 38)	AF recurrence (n = 43)	p value *
APD (mm)	47 ± 5.3	46.8 ± 6.0	47.0 ± 5.1	0.86	44.3 ± 5.9	42.5 ± 5.8	45.9 ± 5.6	< 0.01
CCD (mm)	58.0 ± 9.9	54.6 ± 10.6	59.6 ± 9.2	0.09	56.8 ± 9.5	50.9 ± 8.3	62 ± 7.1	< 0.001
MLD (mm)	44.9 ± 8.5	45.0 ± 9.1	44.8 ± 8.3	0.96	40.2 ± 7.4	38.6 ± 8.1	41.6 ± 6.6	0.06
AV (ml)	76.0 ± 24.0	79.6 ± 28.7	74.2 ± 21.6	0.46	65.6 ± 22.2	57.2 ± 21.2	73.1 ± 20.6	0.001
iAV (ml/m^2^)	42.7 ± 12.9	46.4 ± 14.9	41.0 ± 11.7	0.17	37.0 ± 13.9	32.4 ± 12.1	41.1 ± 14.2	< 0.01
AA (cm^2^)	28.5 ± 9.3	30.3 ± 10.9	27.7 ± 8.5	0.35	23.8 ± 6.6	21.6 ± 5.9	25.7 ± 6.6	< 0.01
AAV (cm/s)	19.5 ± 5.0	22.1 ± 5.5	18.3 ± 4.3	0.01	19.9 ± 5.0	21.8 ± 5.5	18.3 ± 3.9	< 0.01

The continuous variables were described as mean ± standard deviation. *PnVAF* persistent non valvular atrial fibrillation, *LSPnVAF* long standing persistent non-valvular atrial fibrillation, *APD* antero-posterior diameter, *CCD* cephalocaudal diameter, *MLD* medio-lateral diameter, *AV* atrial volume, *iAV* indexed atrial volume, *AA* atrial area, *AAV* atrial appendage velocity

* Student´s *t* test

No differences were observed in the measurements of ventricular parameters between subjects with AF recurrence versus sinus rhythm both in subjects with PnVAF and in those with LSPnVAF (see Table 3).

**Table 3:** Left ventricular echocardiographic characteristics

	PnVAF (n = 50)				LSPnVAF (n = 81)			
	Total (n = 50)	Sinus Rhythm (n = 16)	AF Recurrence (n = 34)	p value *	Total (n = 81)	Sinus Rhythm (n = 38)	AF recurrence (n = 43)	p value *
LVEDD (mm)	45.8 ± 4.6	45 ± 4.6	46.1 ± 4.6	0.41	44.7 ± 4.3	44.9 ± 3.9	44.5 ± 4.6	0.63
LVESD (mm)	28.6 ± 4.4	28.7 ± 5.2	28.8 ± 4.1	0.64	28.2 ± 4.6	28.2 ± 4.1	28.2 ± 5.0	0.96
ST (mm)	12.6 ± 1.3	12.6 ± 1.5	12.7 ± 1.2	0.84	11.9 ± 1.3	11.6 ± 1.5	12.1 ± 1.1	0.11
PWT (mm)	12.4 ± 1.4	12.3 ± 1.5	12.4 ± 1.3	0.93	11.6 ± 1.5	11.2 ± 1.6	11.9 ± 1.2	0.07
LVSV (ml)	31.1 ± 10.1	32 ± 12.3	30.7 ± 9.1	0.69	31.5 ± 11.5	31.1 ± 10.2	31.9 ± 12.6	0.77
EF (%)	67.4 ± 5.7	67 ± 6.7	67 ± 5.3	0.76	66.2 ± 6.4	65.8 ± 6.4	66.5 ± 6.5	0.63

The continuous variables were described as mean ± standard deviation

*PnVAF* persistent non-valvular atrial fibrillation, *LSPnVAF* long standing persistent non-valvular atrial fibrillation; *LVEDD*, left ventricular end diastolic diameter, *LVESD* left ventricular end systolic diameter, *ST* septal thickness, *PWT* posterior wall thickness, *LVSV* left ventricular systolic volume, *EF* ejection fraction

*Student’s *t* test

Regarding the diastolic function, when compared to the sinus rhythm subjects, the group with AF recurrence in PnVAF showed greater E-wave velocity (106.5 ± 21.3 vs. 85.4 ± 18.4 cm/s; p = 0.001); we observed no differences in DT, IVRT, S-wave velocity, E/e’ ratio and PASP. In the LSPnVAF the group of AF recurrence showed greater E-wave velocity (95.7 ± 20.1 vs. 85.4 ± 18.9 cm/s; p = 0.02), E/e’ ratio (12.9 ± 3.7 vs. 10.9 ± 2.7; p < 0.01) and PASP (39.2 ± 6.3 vs. 35.9 ± 6.5 mm Hg; p = 0.02) vs those in sinus rhythm and we observed no differences in DT, IVRT and S-wave velocity by mitral annulus Doppler tissue (see Table 4).

**Table 4:** Echocardiographic characteristics by Doppler

	PnVAF (n = 50)				LSPnVAF (n = 81)			
	Total (n = 50)	Sinus rhythm (n = 16)	AF recurrence (n = 34)	p value *	Total (n = 81)	Sinus rhythm (n = 38)	AF recurrence (n = 43)	p value*
E (cm/s)	99.7 ± 22.5	85.4 ± 18.4	106.5 ± 21.3	0.001	90.9 ± 20.1	85.4 ± 18.9	95.7 ± 20.1	0.02
DT (ms)	228.6 ± 62.5	232.8 ± 70.4	226.6 ± 59.5	0.74	215.5 ± 56.4	222.4 ± 58.4	209.5 ± 54.5	0.30
IVRT (ms)	117.1 ± 17.9	123.3 ± 14.6	114.1 ± 18.8	0.09	111.2 ± 18.3	110.5 ± 17.8	111.9 ± 19	0.73
S (cm/s)	6.4 ± 1.0	6.3 ± 0.9	6.4 ± 1.1	0.78	7.2 ± 2.0	7.4 ± 2.1	7.1 ± 1.9	0.42
E/e´ ratio	13.6 ± 4.3	11.9 ± 3.1	14.4 ± 4.7	0.05	12.0 ± 3.4	10.9 ± 2.7	12.9 ± 3.7	< 0.01
PASP (mm Hg)	37.5 ± 5.8	36.5 ± 4.5	38.0 ± 6.3	0.38	37.7 ± 6.6	35.9 ± 6.5	39.2 ± 6.3	0.02

The continuous variables were described as mean ± standard deviation

*PnVAF* persistent non-valvular atrial fibrillation, *LSPnVAF* long standing persistent non-valvular atrial fibrillation, *LVEDD* left ventricular end diastolic diameter, *LVESD* left ventricular end systolic diameter, *E* E wave, *DT* deceleration time, *IVRT* isovolumetric relaxation time, *S* S wave velocity, *E/E’* ratio E velocity to e’ velocity ratio, *PASP* pulmonary artery systolic pressure

*Student’s *t* test or U Mann–Whitney according to their distribution

The GPALS was 12.1 ± 4.5% in the subjects with PnVAF and 13.6 ± 8.7% in subjects with LSPnVAF (p = ns). GPALS quantification showed good reproducibility: intraclass correlation coefficient 0.95 (95% CI 0.83–0.99, p < 0.001) and 0.92 (95% CI 0.88–0.98, p < 0.001) for intraobserver and interobserver, respectively.

**Fig. 2:**
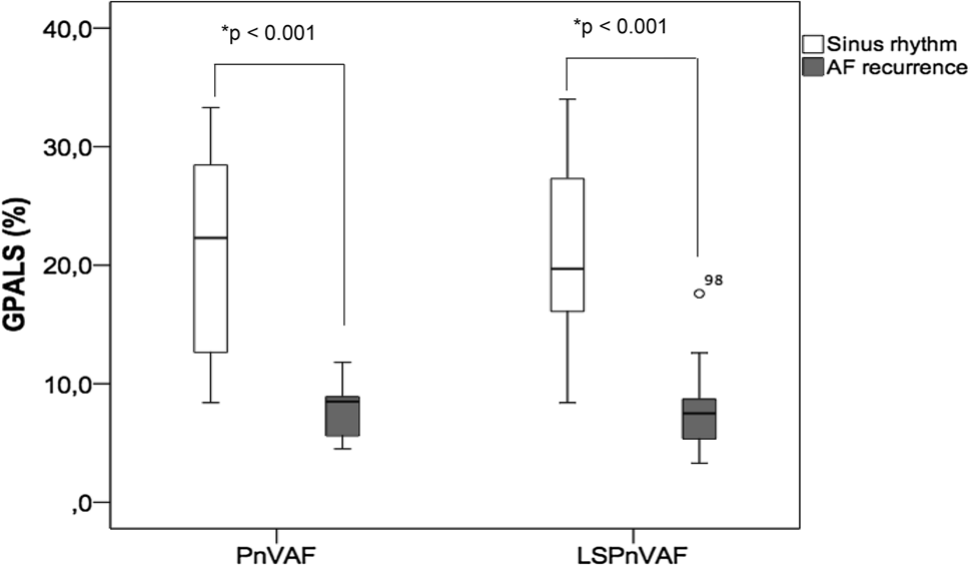
Atrial deformation according to the presence or absence of recurrence after cardioversion, GPALS is lower in subjects with recurrence versus subjects who remained in sinus rhythm in both groups of AF. *U Mann–Whitney. *GPALS* global peak atrial longitudinal strain, *PnVAF* persistent non-valvular AF, *LSPnVAF* long standing persistent non-valvular AF

Figure 2 shows that the subjects with recurrence in PnVAF had a lower GPALS 7.8 ± 2.0% vs. 21.2 ± 8.9% (p < 0.001) and the same phenomenon was observed in LSPnVAF with GPALS 7.3 ± 2.7% versus 20.7 ± 7.6% in AF recurrence vs sinus rhythm respectively (p < 0.001).

**Fig. 3:**
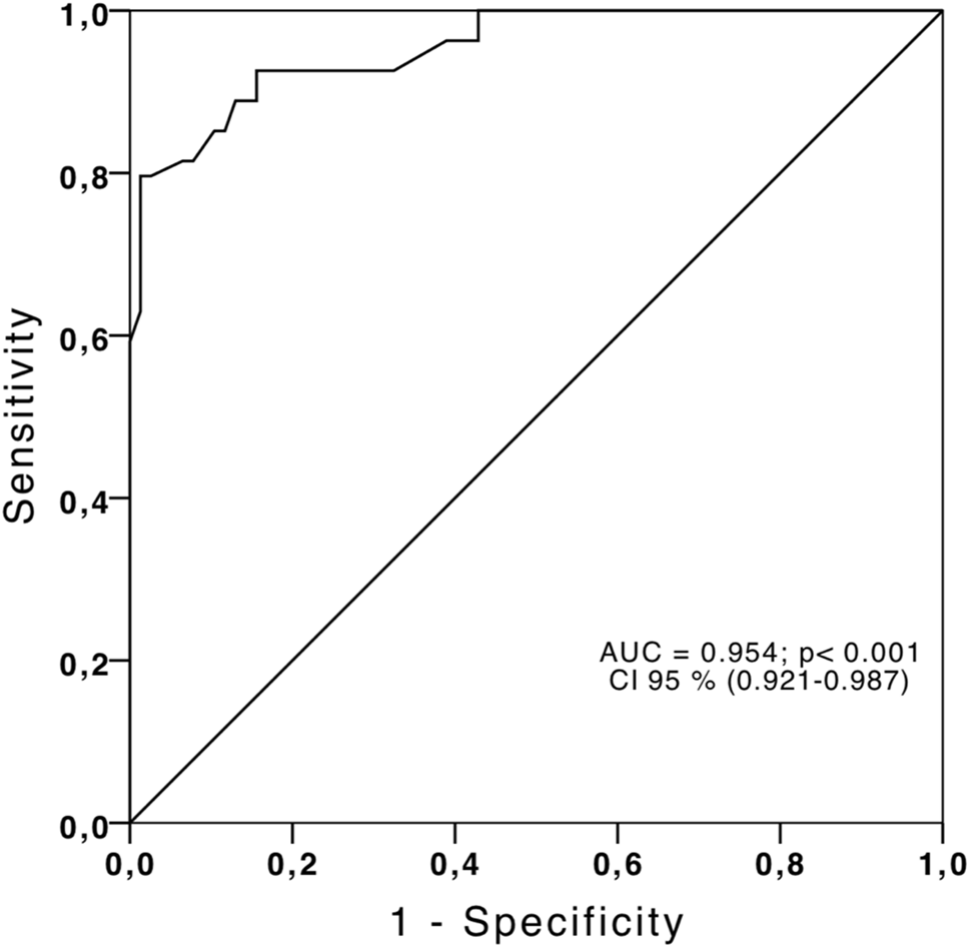
ROC curve for GPALS and risk of recurrence after cardioversion in subjects with PnVAF and LSPnVAF. A cut-point of GPALS ≤ 10.75% identifies recurrence with S 85%, E 99%, PPV 85%, NPV 90%, LR+ 8.5 and LR− 0.17. *GPALS* global peak atrial longitudinal strain, *PnVAF* persistent non-valvular AF, *LSPnVAF* long standing persistent non-valvular AF, *PPV* positive predictive value, *NPV* negative predictive value, *LR+* positive likelihood ratio, *LR−* negative likelihood ratio

**Fig. 4:**
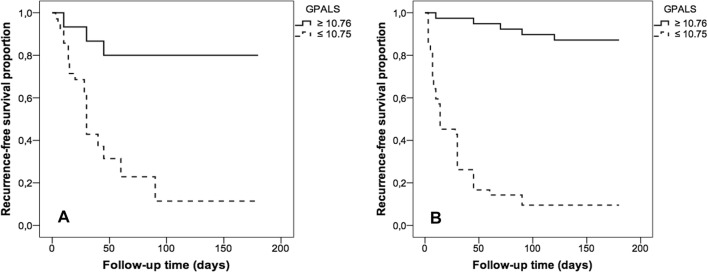
Recurrence-free survival curve of subjects with PnVAF (**a**) and LSPnVAF (**b**) according to the GPALS cut-point obtained by Youden index. The subjects with GPALS ≤ 10.75% have greater recurrence at 6 months versus subjects with GPALS ≥ 10.76%. *PnVA*F persistent non-valvular AF, *LSPnVAF* long standing persistent non-valvular AF, *GPALS* global peak atrial longitudinal strain

According to the Youden index (J) in the ROC curve of the GPALS for recurrence risk, we obtained a cut value of ≤ 10.75%, sensitivity 85%, specificity 99%, positive predictive value 85%, negative predictive value 90%, and finally a LR+ 8.5, and LR− 0.17 (Fig. 3). The multivariate Cox proportional hazards regression analysis included the following variables: age, diabetes mellitus, weight, atrial diameters, volumes, area, velocity of left atrial appendage, E-wave velocity, E/e′ ratio and GPALS. For the PnVAF group only GPALS ≤ 10.75% HR 8.89 [(2.2–35.7), p < 0.01] remains as independent predictor of AF recurrence at 6 months; for LSPnVAF group only age HR 1.039 [(1.007–1.071), p = 0.01], and GPALS ≤ 10.75% HR 28.1 [(7.2–109.1), p < 0.001], remains as independent predictors of AF recurrence at 6 months. The event-free survival curve (Fig. 4) showed that the GPALS ≤ 10.75% cut-point discriminates subjects with sinus rhythm vs those with recurrence at 180-day follow-up in PnVAF and LSPnVAF (p < 0.001).

## Discussion

Nowadays, there is a marked tendency to choose the rhythm control strategy in subjects with symptomatic AF through cardioversion or treatments like ablation. There currently are few studies that include subjects with LSPnVAF [8–14]. Cardioversion brings benefits such as: organization of the atrial activation/contraction and a sequence of normal atrioventricular activation, contribution to atrial contraction with 25–30% of cardiac output, proper functioning of the atrioventricular valves, embolism risk reduction, and reversal remodeling [11]. Nevertheless, there still exists controversy when defining who the best candidates for elective cardioversion are since up to 60% of recurrence [24] has been reported, a fact which is consistent with the findings in this study.

Reports in the literature establish as predictors of recurrence factors such as: age, absence of treatment with antiarrhythmic drugs, use of ACE inhibitors, ARBs and spironolactone; evolution time of arrhythmia, chronic pneumopathy, left atrial diameter > 40 mm, appendage ejection fraction < 30%, appendage emptying flow velocity, and indexed atrial volume, among others [24–33]. In a population of subjects with non-valvular AF, Ahmed et al. [34] found greater values of diameters and atrial volumes in subjects with recurrence. Our results are similar: the subjects with recurrence showed greater atrial diameters, areas and volumes, as well as a lower appendage velocity versus those subjects in sinus rhythm at 6 months. However, we detected no differences regarding age, anti-remodeling drugs, and evolution time of arrhythmia.

One of the proposals that explains recurrence is supported by atrial remodeling as a phenomenon of adaptive regulation with multifactor origin that conditions metabolic, functional, electrical, structural, and neurohumoral changes associated to arrhythmia maintenance and that lead to fibrosis [15, 16, 20–22]. The conventional clinical and echocardiography predictors are associated to recurrence, but they are unable to discriminate the degree of atrial fibrosis. In this opportunity niche, speckle tracking plays a fundamental role since it allows measuring the deformation of the atrial muscle tissue and is indirectly a fibrosis surrogate marker: the greater the deformation, the lower the fibrosis and vice versa [36–42].

The literature points out that, in subjects with AF, GPALS is decreased. Kuppahally et al. [43] reported values of 27 ± 15% in the medio-septal region and 35 ± 18% in the medio-lateral region, as well as an inverse correlation between the degree of fibrosis and atrial deformation (r = −0.5, p < 0.003). We observed that the recruited subjects had a GPALS of 12.1 ± 4.5% in the subjects with PnVAF and 13.6 ± 8.7% in subjects with LSPnVAF. This may be lower than that reported by Kuppahally et al. [43] since we reported the value of global deformation and only included subjects with non-valvular AF and arrhythmia of longer duration.

Dell’Era et al. [45] studied a group of 130 subjects with persistent AF who were taken to electrical cardioversion; 1 month after follow-up, there was 44% of recurrence. In the patients with recurrence, the researchers found a GPALS of 11.36 ± 5.19 versus 17.15 ± 7.5% in the sinus group (p < 0.001). We found a GPALS of 7.8 ± 2.0% vs. 21.2 ± 8.9% (p < 0.001) in subjects with PnVAF and GPALS 7.3 ± 2.7 versus 20.7 ± 7.6 (p < 0.001) in LSPnVAF when comparing AF recurrence vs sinus rhythm respectively. The differences found in the GPALS determination between Dell’Era et al. [45] and our study may be due to the fact that the AF duration in the patients included in this study was greater while the AF was of non-valvular etiology.

Motoki et al. [46] reported that a group of subjects with persistent and paroxysmal AF of multiple etiologies treated with transcatheter ablation had 42% of recurrence at 8-month follow-up. Using the GPALS cut-point 23.2%, they predicted arrhythmia recurrence with sensivity of 76% and specificity of 66%. In subjects with non-valvular AF taken to elective electrical cardioversion, we found that a GPALS cut-point ≤ 10.75% has 85% of sensitivity, 99% of specificity, 85% PPV, 90% NPV, LR+ 8.5 and LR− 0.17. We also found that this cut-point allows identifying recurrence risk at 6 months with HR 8.89 [(2.2–35.7), p < 0.01] for PnVAF and HR 28.1 [(7.2–109.1), p < 0.001] for LSPnVAF. The cut-point detected in our population is slightly lower than the one reported by Motoki et al. [46]. This is probably due to the selection criteria given the fact that we included subjects with non-valvular AF whose evolution time has an ample range; this situation probably favored a greater remodeling and fibrosis.

It is important to say that, in the subjects with arrhythmia recurrence, we observed no differences according to the type of AF (PnVAF vs. LSPnVAF) in terms of diameters, areas and atrial volumes, diastolic function parameters or atrial deformation values. This is consistent with the reports found in literature because these subjects supposedly have greater fibrosis, something that is evident in their values of GPALS 7.8 versus 7.3% (p = ns), respectively. In contrast, in the group of subjects that remained in sinus rhythm at six months, the ones with PnVAF showed greater diameters, areas and atrial volumes when compared with the subjects with LSPnVAF. This finding suggests that the atria of patients with AF of shorter evolution time are more compliant due to a lower degree of fibrosis, considering the difference in the GPALS values. Even though there is a tendency among the subjects with persistent AF to have more deformation, we did not detect a significant statistical difference due to the sample size of recruited subjects.

Taking into account the reference framework laid by Kuppahally et al. [43] and Cameli et al. [44], we can infer that our results suggest that the patients with recurrence have a greater degree of atrial fibrosis (remodeling) evidenced by the reduction in the GPALS as a surrogate marker. This fact is closely related to the physiopathology reported in literature as part of the genesis and maintenance of arrhythmia when creating multiple reentry circuits or depolarization fronts that undergo fractionation [47, 48].

We consider that one of the limitations our study has is that the duration of the arrhythmia was determined according to the information provided by the participants of the study, since there is currently no method that allows defining the exact time the arrhythmia started with precision. Therefore, it might be possible that some patients were not properly classified. Nevertheless, the cut-point of GPALS ≤ 10.75% predicts recurrence in the analysis of all the subjects and in the analysis by AF group.

## Conclusions

In subjects with PnVAF and LSPnVAF taken to elective cardioversion, a cut-point of the GPALS ≤ 10.75% predicts arrhythmia recurrence at 6 months after electrical cardioversion.
